# Possible effects of global environmental changes on Antarctic benthos: a synthesis across five major taxa

**DOI:** 10.1002/ece3.96

**Published:** 2012-02

**Authors:** Jeroen Ingels, Ann Vanreusel, Angelika Brandt, Ana I Catarino, Bruno David, Chantal De Ridder, Philippe Dubois, Andrew J Gooday, Patrick Martin, Francesca Pasotti, Henri Robert

**Affiliations:** 1 Marine Biology Department, Ghent UniversityKrijgslaan 281 S8, 9000 Ghent, Belgium; 2 Zoological Museum Hamburg, University of HamburgMartin-Luther-King-Platz 3, 20146 Hamburg, Germany; 3 Marine Biology Laboratory, Université Libre de BruxellesCP160/15, Avenue F.D. Roosevelt 50, 1050 Brussels, Belgium; 4 Biogeosciences, The University of BurgundyUMR CNRS 5561, Bd Gabriel 6,21000 Dijon, France; 5 Ocean Biogeochemistry & Ecosystems Research Group, National Oceanography CentreEuropean Way, Southampton SO14 3ZH, United Kingdom; 6 Royal Belgian Institute of Natural SciencesRue Vautier 29, 1000 Brussels, Belgium

**Keywords:** Amphipoda, Echinoidea, Foraminifera, global climate change, Isopoda, Nematoda, Southern Ocean, zoobenthos

## Abstract

Because of the unique conditions that exist around the Antarctic continent, Southern Ocean (SO) ecosystems are very susceptible to the growing impact of global climate change and other anthropogenic influences. Consequently, there is an urgent need to understand how SO marine life will cope with expected future changes in the environment. Studies of Antarctic organisms have shown that individual species and higher taxa display different degrees of sensitivity to environmental shifts, making it difficult to predict overall community or ecosystem responses. This emphasizes the need for an improved understanding of the Antarctic benthic ecosystem response to global climate change using a multitaxon approach with consideration of different levels of biological organization. Here, we provide a synthesis of the ability of five important Antarctic benthic taxa (Foraminifera, Nematoda, Amphipoda, Isopoda, and Echinoidea) to cope with changes in the environment (temperature, pH, ice cover, ice scouring, food quantity, and quality) that are linked to climatic changes. Responses from individual to the taxon-specific community level to these drivers will vary with taxon but will include local species extinctions, invasions of warmer-water species, shifts in diversity, dominance, and trophic group composition, all with likely consequences for ecosystem functioning. Limitations in our current knowledge and understanding of climate change effects on the different levels are discussed.

## Introduction

The Southern Ocean (SO) covers about 34.8 million km^2^ and the Antarctic contains roughly 11% of the world's continental-shelf area (Zwally et al. 2002). This vast region already harbors a significant share of the planet's marine diversity (roughly 5 % based on currently officially described marine species—based on Register of Antarctic Marine Species and World Register of Marine Species) (Clarke and Johnston 2003; Barnes and Peck 2008; Barnes et al. 2009b; Brandt et al. In Press, 2007). However, conservative estimates suggest that a vast number of Antarctic benthic species still remain undiscovered due to chronic undersampling of seafloor habitats, lack of specialists studying important taxa (Gutt et al. 2004; Brandt et al. 2007; Griffiths 2010; Griffiths et al. 2011), and possible cryptic species neglect (Clarke and Johnston 2003; Held 2003; Gutt et al. 2004; Held and Wägele 2005; Raupach and Wägele 2006; Linse et al. 2007; Raupach et al. 2007a; Havermans et al. 2011). Compared to shallower Antarctic waters, knowledge of SO deep-sea benthic diversity remains even more limited, although available morphological and molecular data give evidence for highly diverse communities (Brandt et al. in Press, 2007; Raupach et al. 2007a). The increasing need for an exhaustive inventory of marine Antarctic biodiversity has stimulated the creation of international concerted database initiatives based on open-access policies (e.g., SCAR MarBIN, ANTABIF, Polar Information Commons, [Danis and Griffiths 2009; Griffiths et al. 2011]), and programmes and projects (e.g., Census of Antarctic Marine Life, ANDEEP, PolarBoL as part of the International Barcode of Life Project) with the aim to aggregate all currently available SO biodiversity data and create opportunities to fill the gaps in our knowledge. The currently available information on SO biodiversity has enabled scientists to start describing and explaining biodiversity patterns and test biogeographical and macroecological hypotheses. These efforts have contributed significantly to our understanding of the underlying processes that drive and maintain Antarctic benthic diversity (Clarke and Johnston 2003; Brandt and Ebbe 2009), but they have also shown that questions regarding the origin, diversification, and extinction of Antarctic benthic species cannot be answered by studying one taxonomic group (Clarke and Johnston 2003). Different benthic groups are shaped by their individual evolutionary history and reproductive strategies, reflecting different responses to the tectonic, climatic, and oceanographic changes of the past. Notwithstanding the differences in responses to historical changes in their environment, Antarctic benthic taxa are generally perceived as vulnerable to environmental shifts, notably in temperature and pH (acidification) (Orr et al. 2005; Barnes and Peck 2008). Yet, the sensitivity or vulnerability of Antarctic benthos to these environmental changes may vary markedly depending on which level of faunal organization is considered (from genes to individuals, populations, species, communities, and ecosystems). Physiological responses of individuals to small temperature fluctuations, for instance, may reveal high sensitivity (Peck et al. 2004) while the resulting community or ecological response to such temperature changes may be less evident (Aronson et al. 2009). Similarly, physiological or ecological responses to changes in temperature do not automatically entail a response to other environmental shifts. Crucial to understanding benthic responses to environmental change are the complex chains of functional interactions between benthic organisms, and their diverse ecologies. Despite the expansion in our knowledge of individual species responses to climate-induced changes, there is still little understanding of these effects at community levels and the underlying mechanisms that control these effects. Recently, more integrated research approaches have tried to fill in the gap of our ecological and functional understanding of SO biota, including its relation with the physicochemical environment (projects such as ANDEEP-SYSTCO [Brandt et al. 2011], FOODBANCS [Smith and DeMaster 2008], Palmer LTER [Ducklow 2008], and SAZ-Sense [Bowie et al. 2011]), but many more questions remain unanswered. Moreover, ecosystem changes in response to climate change are poorly documented because there is a lack of understanding of the processes linking the different levels of faunal organization and the variable effect of climatic changes on these different life stages.

**Figure 1:**
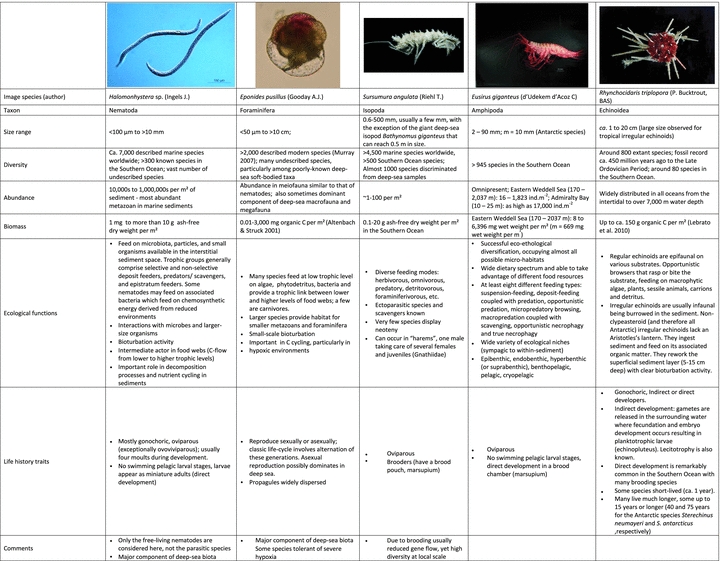
Important characteristics of the five taxa reviewed in this study.

For an improved assessment of the effect of environmental change on diversity, physiology and ecological functioning of Antarctic marine benthos, it is essential to consider a range of different taxa. In this study, we focus on the Foraminifera, Nematoda, Isopoda, Amphipoda, and Echinoidea, representing the meiofauna (32–1000 µm), the macrofauna (>1 mm), and the megafauna (>10 cm, visible with underwater photography). A summary of important characteristics of these taxa are given in Figure 1. These five groups are highly diverse and include many of the more than 4000 described Antarctic benthic species (Clarke and Johnston 2003). Moreover, they are ecologically important in terms of biomass (Brey and Gutt 1991; Barnes and Brockington 2003), their role in biogeochemical cycles (C and N) (Danovaro et al. 1999; Moodley et al. 2002; Nomaki et al. 2005; Woulds et al. 2007; Gooday et al. 2008; Lebrato et al. 2010; Piña-Ochoa et al. 2010), and their trophic role in benthic ecosystems (De Ridder and Jangoux 1982; De Ridder and Lawrence 1982; Gooday et al. 1992; Danovaro et al. 1999; Dauby et al. 2001; Suhr et al. 2003; De Broyer et al. 2004; Nomaki et al. 2008). They are also characterized by different biogeographic and diversity patterns, modes of speciation, and reproductive and dispersal mechanisms (Watling and Thurston 1989; Brandt 1999; De Broyer and Rauschert 1999; Raupach and Wägele 2006; Brandt et al. 2007; Murray 2007; Malyutina and Brandt 2007; Pawlowski et al. 2008; Choudhury and Brandt 2009; Lecroq et al. 2009). These five taxa are key contributors to the diversity and functioning of SO benthic ecosystems, but they are of course not the only benthic components playing an important role. Important players in SO benthic ecosystems also include holothurians (Gutt 1991a, b; Gutt and Piepenburg 1991; Mincks et al. 2008; O'Loughlin et al. 2011), bivalves (Brey et al. 1996, 2011; Linse et al. 2006a, b; Brandt et al. 2009), polychaetes (Glover et al. 2008; Brandt et al. 2009; Neal et al. 2011; Würzberg et al. 2011b), sponges (Janussen and Tendal 2007; Bell 2008; Amsler et al. 2009), and prokaryotes (Tindall 2004), among many others. While it is crucial that multiple taxa are considered in assessing climate change effects, inclusion of all taxa in this study is not feasible. Comparable review studies on other important SO taxa are urgently needed to come to a better understanding of climate change effects and responses of SO biota.

Here, a review is provided on the current knowledge on the ability of these five taxa to cope with the most severe climate-related environmental changes (warming, acidification, ice retreat, food quantity and quality, oxygen, and salinity) from the individual to the taxon-specific community level. First, we give an overview of major expected changes in the Antarctic marine environment from the coastal zones to the deep sea, focusing on the global change induced drivers that are expected to impact the Antarctic zoobenthos. We also present taxon-specific sensitivity tables, based on Antarctic and non-Antarctic literature. These identify the known responses of the selected taxa from species to the taxon-specific community level to the specific environmental changes. Limitations and gaps of our current knowledge and understanding of climate change on the different levels of biological organization are discussed, and suggestions to address the unknowns are given.

## Global change-induced drivers for Antarctic benthic faunal change

**Figure 2:**
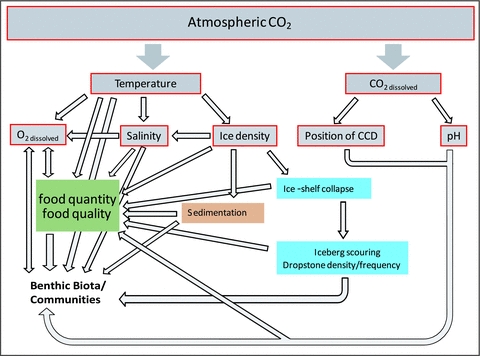
Flow chart of the main effects climate change caused in the marine environment, indicating a cascade of effects that will ultimately have an effect on the benthic biology. Red-framed boxes indicate interacting physico-chemical variables that act to change the environmental settings and can have an effect on the benthic biota or communities. Blue, brown, green colored boxes are factors that are affected by the physico-chemical variables, which may interact with each other and cause a type of disturbance to the benthic biota/communities.

Climate change over the past few decades has already caused significant shifts in marine and terrestrial ecosystems (Hughes 2000; Walther et al. 2002; Thomas et al. 2004). Marine species are affected by physical and biochemical alterations of our oceans caused by increasing emissions and rising temperatures. Antarctic ecosystems, particularly those around the Antarctic Peninsula, a region which is experiencing one of the fastest rates of regional climate change on Earth (Turner et al. 2009), are particularly vulnerable and sensitive. Continued warming together with increasing CO_2_ concentrations in the SO is causing a cascade of environmental effects with far-reaching consequences for the benthic fauna (Fig. 2, flow chart).

Since 2000, global anthropogenic CO_2_ emissions have been rising at unprecedented rates and exceed worst-case scenarios developed by the Intergovernmental Panel on Climate Change (IPCC's Fourth Assessment Report) (Raupach et al. 2007b). As atmospheric CO_2_ concentrations rise, ocean CO_2_ uptake increases and the chemical balance of seawater is disturbed, causing the pH to decrease with a wide range of consequences for marine pelagic and benthic life and ecosystems (Gattuso and Hansson 2011). Consequently, the production of biogenic calcium carbonate (both aragonite and the less soluble calcite) becomes more and more difficult for certain marine organisms (Orr et al. 2005; Gazeau et al. 2007; Kroeker et al. 2010). Increasing temperatures and CO_2_ solubility will cause the calcium carbonate saturation horizon and the CCD (calcium carbonate compensation depth) to shoal, hence exposing organisms to new saturation states that may impact calcification processes. SO waters experience faster acidification rates because low surface temperatures increase CO_2_ solubility and greater upwelling of deep water that contains high levels of CO_2_ due to organic matter demineralization (Guinotte and Fabry 2008). Despite this, SO studies on acidification remain sparse compared to other regions worldwide. Models predict that by 2100, the entire SO water column will become undersaturated with respect to aragonite (Orr et al. 2005; Matear and Lenton 2008), and as early as 2050 for surface waters (Fabry et al. 2009). Recent results indicate that this has already occurred in surface and shallow subsurface waters in some areas of northern polar seas (Fabry et al. 2009). The calcite horizon will remain at ∼2200-m water depth, although in the Weddell Sea calcite undersaturation may reach the surface waters (Orr et al. 2005). Since preindustrial times, the average surface seawater pH has already been reduced by approximately 0.1 units, while projected pH changes in the SO surface waters by 2100 range from 0.3 to 0.5 units (Caldeira and Wickett 2003; Orr et al. 2005; McNeil and Matear 2008). The predicted decrease of pH and changes in CO_2_ solubility may impede calcification and other physiological processes such as growth and respiration (Pörtner et al. 2004; Doney et al. 2009; Kroeker et al. 2010). Furthermore, ocean acidification can cause phytoplankton community shifts, which will influence community structure of the higher trophic levels that are reliant on the phytoplankton (Hays et al. 2005; Smith et al. 2008a). Acidification may also influence the activity of bacteria (which produce CO_2_) and zooplankton (which consume phytoplankton), resulting in changes in the structure and functioning of the marine ecosystem as a whole (Pörtner et al. 2004). Marine biota, however, do not respond uniformly to ocean acidification and overall ecosystem responses to acidification will be different from species responses (Caldeira and Wickett 2003; Doney et al. 2009; Kroeker et al. 2010). Moreover, current knowledge about sedimentary biogeochemical processes under acidified conditions and subsequent effects on benthic biology is insufficient to infer ecosystem-scale effects.

While global oceanic uptake of anthropogenic CO_2_ is estimated at about 25–40% (Matear and Hirst 1999; Takahashi et al. 2009), the SO below 50° S is responsible for only 4–9% of global anthropogenic CO_2_ storage (Sabine et al. 2004; Takahashi et al. 2009). Although air–sea CO_2_ fluxes into the SO are relatively high, its capacity as a sink is limited because most CO_2_ is transported northward through deep-water thermohaline circulation (Caldeira and Duffy 2000). Various climate change studies based on the carbon-climate system predict a decrease in efficiency of the oceans as a sink for anthropogenic CO_2_ (Matear and Hirst 1999; Plattner et al. 2001; Canadell et al. 2007; Le Quéré et al. 2007; Sabine and Tanhua 2010), Positive feedback caused by increasing sea surface temperatures, changes in carbonate chemistry and ocean circulation will outweigh negative feedback effects (e.g., increased primary production), hence reducing global oceanic CO_2_ uptake by up to nearly 30% during the 21st century (Matear and Hirst 1999). This scenario applies particularly in the case of the SO, where the impact of warming, transport processes, and biological effects is greater than in other oceans (Sabine et al. 2004). This reflects the sensitivity of the SO to changes in stratification of the water column and the fact that deep mixing is normally able to encompass the vast volume of deep water that holds excess biogenic carbon (Sarmiento and Orr 1991). Although the efficiency with which the SO takes up CO_2_ under climate change forcing is still debated (Le Quéré et al. 2007; Matear and Lenton 2008), it will certainly decrease if the present climate trends continue (Le Quéré et al. 2007; Matear and Lenton 2008).

Besides reducing CO_2_ solubility in sea water, rising temperatures may have direct impacts on the physiology of stenothermal organisms (Peck 2005) as well as on the extent of sea ice and hence the life history and biology of many species (Barnes and Peck 2008). As well as affecting the physiology, phenology, and ontogeny of species, temperature increases may also modify their geographic distributions and alter biological invasion processes (Walther et al. 2009). Moderate temperature shifts are expected within the next 100 years; models suggest a 0.5–1.0°C rise in SO surface waters in summer, with local temperature increases up to 2.0°C, but winter temperatures will only increase by a maximum of 0.5°C (Turner et al. 2009). Regardless of seasons, bottom waters from the surface down to 4000 m depth are expected to warm on average by around 0.25°C, with higher temperatures possible at deeper shelf depths (Barnes et al. 2009a). At abyssal depths, the temperature change seems small, but the compound effects of temperature and reduction or decoupling of the pelago-benthic relationship as a consequence of biogeochemical changes at the sea surface (including rising sea surface temperatures, thermal stratification, and reduced nutrient upwelling) may alter deep-sea benthic assemblages drastically (Smith et al. 2008a).

The effect of rising atmospheric and sea surface temperatures in the Antarctic have already caused significant changes in sea-ice density over the last 50 years (Zwally et al. 2002), especially at the Antarctic Peninsula (Clarke et al. 2007). Recent models predict a reduction in Antarctic sea ice extent of 24–33% (Arzel et al. 2006; Bracegirdle et al. 2008), albeit with considerable regional variation. Numerous Antarctic marine organisms depend on the seasonally dynamic interface between ice and water and small temperature differences can have large effects on this interface and its associated organisms. Free-drifting icebergs can substantially affect the pelagic ecosystem of the SO and can be considered areas of enhanced production and sequestration of organic carbon to the deep sea (Smith Jr et al. 2007; Smith Jr 2011; Smith Jr et al. 2011). Hence, variation in sea-ice density and extent impact not only the ice-associated (sympagic) fauna, such as certain copepods, amphipods, algae, and microorganisms, but also organisms that depend on algal blooms for food (Mincks et al. 2005; Mincks and Smith 2007; McClintic et al. 2008; Smith and DeMaster 2008; Smith et al. 2008b). These include benthic species relying on phytodetritus from the euphotic zone. A southward retreat of sea ice will modify the extent and density of algae blooms with effects down the food web (Smetacek and Nicol 2005; McClintic et al. 2008; Mincks et al. 2008; Wigham et al. 2008; Montes-Hugo et al. 2009). Furthermore, ice melt can lead to a substantial release of ice fauna into the water column, where it may enhance phytoplankton growth (Gradinger 1999) or sink to the sea floor, serving as food for the benthos (Gradinger 2001). In addition, the gradual disintegration of ice shelves will also reveal new habitats for both pelagic and benthic organisms as well as euphotic primary production, which in turn may influence the quality and quantity of food available to the benthos (Thrush et al. 2006; Bertolin and Schloss 2009). Finally, ice shelves may dampen internal waves and tidal amplitudes and attenuate the effect of storm surges and strong winds on local hydrography, especially in shallow waters. A reduction in sea ice extent may therefore lead to increased hydrodynamic disturbance of the benthos.

Rising temperatures will also lead to deglaciation on land and hence increased glacial discharges in the coastal zones. The resulting higher sedimentation rates are likely to have a considerable but localized impact on benthic communities (Barnes et al. 2009a). The large-scale retreat of maritime glaciers and ice shelves (Cook et al. 2005) will also increase the number of floating icebergs in the short term, leading to increased scouring rates and increased drop stone densities (Lee et al. 2001a, b; Gutt and Piepenburg 2003). While iceberg scouring is known to have a detrimental impact on benthic communities in an initial phase with removal of complete faunal assemblages, patterns and mechanisms of recovery are complex and disturbance-rate and spatial-scale dependent. Disturbance caused by drop stones is usually infrequent, small scale and low magnitude, but following major ice-shelf failure drop stone disturbance can be pervasive and change soft sediment habitats fundamentally by partially or completely covering them (Domack et al. 2005). Scouring disturbances are mostly limited to the continental shelf, where it is shallow enough for floating icebergs to impact the seabed—usually less than ca. 500 m (Gutt et al. 1996; Dowdeswell and Bamber 2007), but deeper scours have been observed (Dowdeswell and Bamber 2007), while drop stones effects can also be significant beyond iceberg-scour depths. In the long term, however, ice scour rates, depth of iceberg scours, and drop-stone intensity are expected to decrease as ice sheets and glaciers become thinner and retreat toward land, and the number and size of scouring icebergs that are released into the waters will diminish. On what time scale these shifts are to be expected, however, remains uncertain and depends on the rates of glacial melt. On the other hand, reduced iceberg scouring may result in decline of species diversity by reducing disturbance frequencies (Gutt and Starmans 2001; Johst et al. 2006).

On long time scales, the compounded effects of increased seasonal melting of glaciers, ice sheets and ice shelves, reduced brine rejection, and rising water temperatures are likely to increase freshwater input and reduce salinity along Antarctic coastal waters (Jacobs et al. 2002), especially at the Antarctic Peninsula. However, no large salinity changes are expected during the 21st century, except above 400-m water depth, where it may drop by up to 0.3 units (Barnes et al. 2009a). Surface water freshening can have a wide range of effects on both the water column and the seabed. These include increased stratification of the water column, which will reduce light and oxygen penetration with possibly pervasive biological effects (Barnes et al. 2009a).

Rising temperatures reduce the solubility of oxygen in water, but deoxygenation of Antarctic surface waters solely through increasing temperatures is unlikely to reach levels deleterious for most benthic organisms. However, thermal changes will coincide with enhanced stratification, increased CO_2_ levels, and elevated oxygen demand of organisms, all of which will promote the development of hypoxic zones with potentially harmful impacts on marine ecosystems in the future (Hofmann and Schellnhuber 2009). Furthermore, increased stratification will reduce the flow of dense, oxygen-rich surface waters to the deep sea, reducing oxygen availability in this environment (Matear et al. 2000). Because the Antarctic is the principal source of oxygen-rich waters for the global deep-sea environment, reduced flow—including attenuation of Antarctic Bottom Water and Antarctic Intermediate Water formation (Broecker et al. 1998; Matear et al. 2000; Hofmann and Schellnhuber 2009), combined with reduced bottom-water oxygen concentrations may have far-reaching repercussions for the global marine biota (Hofmann and Schellnhuber 2009; Pörtner 2010).

Rising temperatures (together with limited salinity changes) may also affect hydrographic barriers such as the Polar Front in the SO. The Polar Front represents a distinctive biogeographical discontinuity, setting boundaries for faunal exchange mainly in the upper pelagic realm. Such exchanges may be influenced by regional climate change, enabling invertebrate larvae to penetrate further south and threaten Antarctic marine biota (Clarke et al. 2005). However, the considerable temperature changes required to enable the invasive migration of larvae from more northerly locations, and their establishment in the Antarctic, render such threats relatively unlikely (Thatje 2005).

Climate change and its complex and interactive chain of associated effects will influence the physiology, distribution, phenology, and ontogeny of many Antarctic benthic organisms. However, the resulting faunal changes, from the species to the community level, remain poorly quantified and understood. Individual species may appear vulnerable to environmental shifts or regime changes, but communities and ecosystems may be more resilient (Brandt 2005; Clarke et al. 2007). Particularly our lack of conceptual and quantitative knowledge on mechanisms that explain climate change responses (Brown et al. 2011; Russell et al. 2011)—and their translation from within species level across biological systems (Russell et al. 2011)—and the plethora of interactions between the many ecosystem components prevent realistic assessments of ecosystem level responses. Before extrapolation from the individual taxon level to communities and ecosystems is achievable, however, there is the initial need for knowledge on taxon-level responses of different benthic ecosystem components to climate change effects. The insight in how different taxa will respond and a preliminary understanding of how they may interact may provide the framework for ecosystem-level assessments.

Below, we provide an overview of the current knowledge about responses of five important groups of benthic organisms to climate change effects, from effects on individuals, populations, and taxon-specific communities. In order to summarize the impacts and understand the potential consequences, we review the taxa in turn and present corresponding sensitivity tables (Tables 1–5), which summarizes the expected reaction of each taxon to different environmental changes.

**Table 1:** Sensitivity table Foraminifera. Sensitivity of Foraminifera to the main consequences of climate change (Warming, Acidification, Ice coverage, Food, O_2_ concentration, and Salinity). Different levels of biological organization are considered, going from the individual level, over population level, up to community level. Within each level, specific functions were selected to identify impacts. “Nutrition” comprises all the processes of feeding, ingestion, digestion, assimilation, but also energy acquisition and allocation to different growth processes. “Sustenance” relates to all processes affecting the survival or sustainability of the population. Color codes indicate the level of impact (see color code table). “Warming” comprises all temperature effects. “Acidification” relates to the lowering of the pH in the sea water. “Ice scour” comprises the impact of iceberg disturbance, whilst “Ice cover” relates to the decrease of ice shelf coverage and ice shelf collapse and can also be seen as a proxy for food changes. “Food quality” refers to the composition and nature of the food available to the benthic community, whilst “Food quantity” refers to the amount of food available to the benthic community

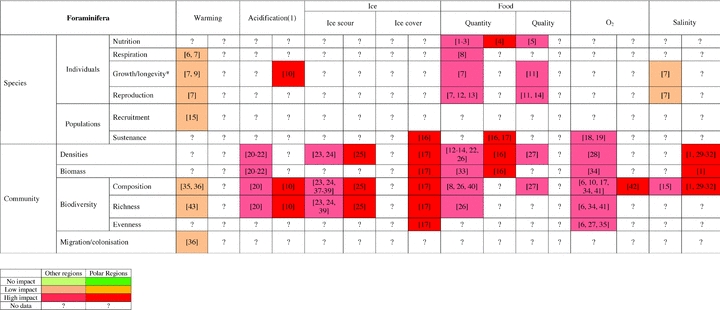

* Including calcification potential

(1) Will affect mainly the calcareous species

References: 1. Korsun 2002; 2. Mojtahid et al. 2011; 3. Nomaki et al. 2005; 4. Bowser et al. 1996; 5. Nomaki et al. 2006; 6. Nomaki et al. 2007; 7. Bradshaw 1961; 8. Linke et al. 1995; 9. Bradshaw 1957; 10. Anderson 1975; 11. Lee 1980; 12. Heinz et al. 2002; 13. Heinz et al. 2001; 14. Lee et al. 1969; 15. Williams 1995; 16. Ahrens et al. 1997; 17. Wollenburg and Mackensen 1998; 18. Woulds et al. 2009; 19. Woulds et al. 2007; 20. Bernhard et al. 2009; 21. Green et al. 1993; 22. Moodley et al. 1993; 23. Langezaal et al. 2003; 24. Langezaal et al. 2004; 25. Lipps and DeLaca 1980; 26. Alve 1995; 27. Fontanier et al. 2005; 28. Schafer et al. 1996; 29. Korsun and Hald 1998; 30. Korsun and Hald 2000; 31. Polyak et al. 2002; 32. Sabbatini et al. 2007; 33. Altenbach and Struck 2001; 34. Gooday et al. 2000; 35. Culver and Buzas 1995; 36. Sen Gupta and Machain–Castillo 1993; 37. Hess et al. 2005; 38. Kaminski 1985; 39. Koho et al. 2007; 40. Altenbach et al. 1999; 41. Gooday et al. 2009; 42. Bernhard 1993; 43. Hayward 2002.

**Table 2:** Sensitivity table Nematoda. Sensitivity of Nematoda to the main consequences of climate change (Warming, Acidification, Ice coverage, Food, O_2_ concentration, and Salinity). Different levels of biological organization are considered, going from the individual level, over population level, up to community level. Within each level, specific functions were selected to identify impacts. “Nutrition” comprises all the processes of feeding, ingestion, digestion, assimilation, but also energy acquisition and allocation to different growth processes. “Sustenance” relates to all processes affecting the survival or sustainability of the population. Color codes indicate the level of impact (see color code table). “Warming” comprises all temperature effects. “Acidification” relates to the lowering of the pH in the sea water. “Ice scour” comprises the impact of iceberg disturbance, whilst “Ice cover” relates to the decrease of ice shelf coverage and ice shelf collapse and can also be seen as a proxy for food changes. “Food quality” refers to the composition and nature of the food available to the benthic community, whilst “Food quantity” refers to the amount of food available to the benthic community

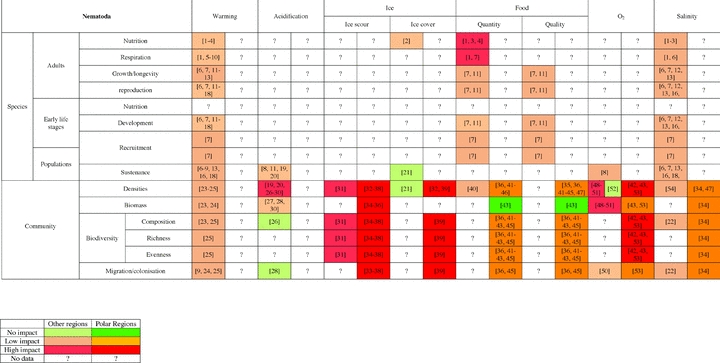

References: 1. Moens and Vincx 2000b; 2. Pascal et al. 2008a; 3. Pascal et al. 2008b; 4. Woombs and Laybournparry 1984; 5. Kim and Shirayama 2001; 6. Warwick 1981; 7. Heip et al. 1985; 8. Wieser et al. 1974; 9. Wieser and Schiemer 1977; 10. Price and Warwick 1980; 11. Vranken et al. 1988; 12. Tietjen and Lee 1972; 13. Tietjen et al. 1970; 14. Vranken and Heip 1986; 15. Heip et al. 1978; 16. Moens and Vincx 2000a; 17. Gerlach and Schrage 1971; 18. Tietjen and Lee 1977; 19. Takeuchi et al. 1997; 20. Ishida et al. 2005; 21. Nozais et al. 1999; 22. Forster 1998; 23. Yodnarasri et al. 2008; 24. Danovaro et al. 2001; 25. Danovaro et al. 2004; 26. Fleeger et al. 2006; 27. Barry et al. 2004; 28. Carman et al. 2004; 29. Barry et al. 2005; 30. Kurihara et al. 2007a; 31. Schratzberger and Warwick 1998; 32. Chen et al. 1999; 33. Peck et al. 1999; 34. Urban–Malinga et al. 2005; 35. Urban–Malinga et al. 2004; 36. Urban–Malinga et al. 2009; 37. Lee et al. 2001b; 38. Lee et al. 2001a; 39. Raes et al. 2009a; 40. Smith et al. 2008a; 41. De Mesel et al. 2006; 42. Vanhove et al. 1998; 43. Vanhove et al. 2000; 44. Fabiano and Danovaro 1999; 45. Urban–Malinga and Burska 2009; 46. Skowronski and Corbisier 2002; 47. Alkemade and Vanrijswijk 1993; 48. Neira et al. 2001a; 49. Neira et al. 2001b; 50. Levin et al. 2009; 51. Gutierrez et al. 2008; 52. Cook et al. 2000; 53. Hendelberg and Jensen 1993; 54. Ruso et al. 2007.

**Table 3:** Sensitivity table Amphipoda. Sensitivity of Amphipoda to the main consequences of climate change (Warming, Acidification, Ice coverage, Food). Different levels of biological organization are considered, going from the individual level, over population level, up to community level. Within each level, specific functions were selected to identify impacts. “Nutrition” comprises all the processes of feeding, ingestion, digestion, assimilation, but also energy acquisition and allocation to different growth processes. “Sustenance” relates to all processes affecting the survival or sustainability of the population. Color codes indicate the level of impact (see color code table). “Warming” comprises all temperature effects. “Acidification” relates to the lowering of the pH in the sea water. “Ice scour” comprises the impact of iceberg disturbance, whilst “Ice cover” relates to the decrease of ice shelf coverage and ice shelf collapse and can also be seen as a proxy for food changes. “Food quality” refers to the composition and nature of the food available to the benthic community, whilst “Food quantity” refers to the amount of food available to the benthic community

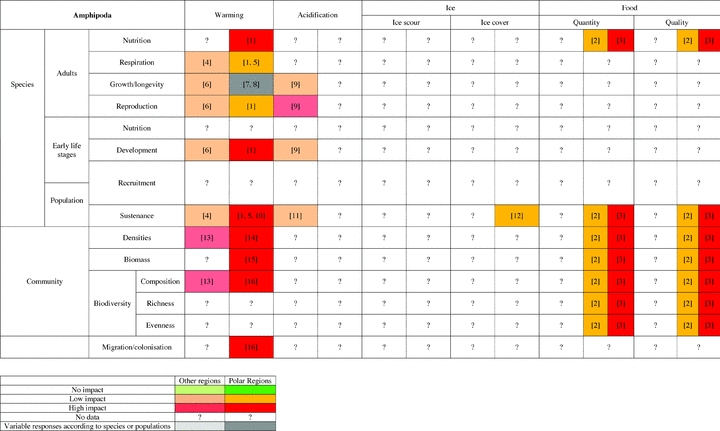

References: 1. Auel and Ekau 2009; 2. Dauby et al. 2001; 3. Nyssen et al. 2005; 4. Lindström and Fortelius 2001; 5. Chapelle and Peck 1999; 6. Maranhão and Marques 2003; 7. Clark et al. 2008; 8. Peck et al. 2009; 9. Egilsdottir et al. 2009; 10. Chapelle 2002; 11. Felten et al. 2006; 12. Hop and Pavlova 2008; 13. Mouritsen et al. 2005; 14. Aronson et al. 2007; 15. Coyle et al.; 16. Barnes et al. 2009b.

**Table 4:** Sensitivity table Isopoda. Sensitivity of Isopoda to the main consequences of climate change (Warming, Acidification, Ice coverage, Food). Different levels of biological organization are considered, going from the individual level, over population level, up to community level. Within each level, specific functions were selected to identify impacts. “Nutrition” comprises all the processes of feeding, ingestion, digestion, assimilation, but also energy acquisition and allocation to different growth processes. “Sustenance” relates to all processes affecting the survival or sustainability of the population. Color codes indicate the level of impact (see color code table). “Warming” comprises all temperature effects. “Acidification” relates to the lowering of the pH in the sea water. “Ice scour” comprises the impact of iceberg disturbance, whilst “Ice cover” relates to the decrease of ice shelf coverage and ice shelf collapse and can also be seen as a proxy for food changes. “Food quality” refers to the composition and nature of the food available to the benthic community, whilst “Food quantity” refers to the amount of food available to the benthic community

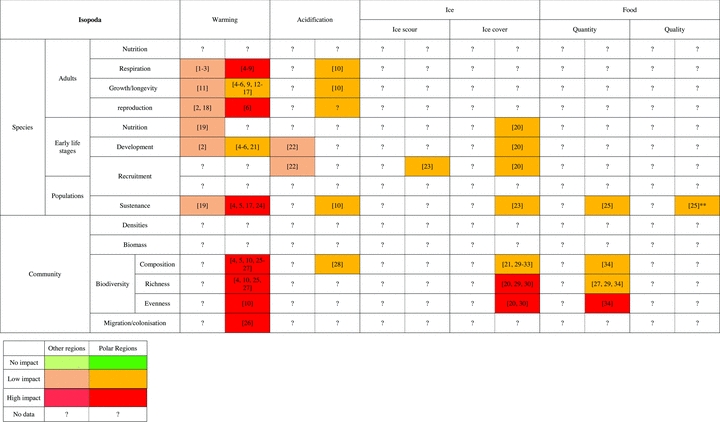

References: 1. Lapucki and Normant 2008; 2. Normant et al. 1998; 3. Hagerman and Szaniawska 1990; 4. Aronson et al. 2007; 5. Frederich et al. 2001; 6. Jokumsen et al. 1981; 7. Luxmoore 1984; 8. Peck et al. 2009a; 9. White 1975; 10. Barnes and Peck 2008; 11. Normant and Szaniawska 1993; 12. Luxmoore 1982; 13. Peck et al. 2009; 14. Pörtner et al. 2007; 15. Wägele 1990; 16. White 1984; 17. Young et al. 2006; 18. Willows 1987; 19. Normant and Szaniawska 1996; 20. Gutt et al. 2011; 21. Pearse et al. 2009; 22. Dupont and Thorndyke 2009; 23. Barnes and Conlan 2007; 24. Janecki et al. 2010; 25. Kaiser and Barnes 2008; 26. Barnes et al. 2009b; 27. Brandt et al. 2007; 28. Orr et al. 2005; 29. Arntz and Clarke 2002; 30. Clarke and Arntz 2006; 31. Clarke et al. 2007a; 32. Clarke et al. 2007; 33. Leese et al. 2008; 34. Arntz et al. 1997.

**Table 5:** Sensitivity table Echinoidea. Sensitivity of Echinoidea to the main consequences of climate change (Warming, Acidification, Ice coverage, Food). Different levels of biological organization are considered, going from the individual level, over population level, up to community level. Within each level, specific functions were selected to identify impacts. “Nutrition” comprises all the processes of feeding, ingestion, digestion, assimilation, but also energy acquisition and allocation to different growth processes. “Sustenance” relates to all processes affecting the survival or sustainability of the population. Color codes indicate the level of impact (see color code table). “Warming” comprises all temperature effects. “Acidification” relates to the lowering of the pH in the sea water. “Ice scour” comprises the impact of iceberg disturbance, whilst “Salinity” relates to the decrease of ice shelf coverage and subsequent salinity changes. “Food quality” refers to the composition and nature of the food available to the benthic community, whilst “Food quantity” refers to the amount of food available to the benthic community

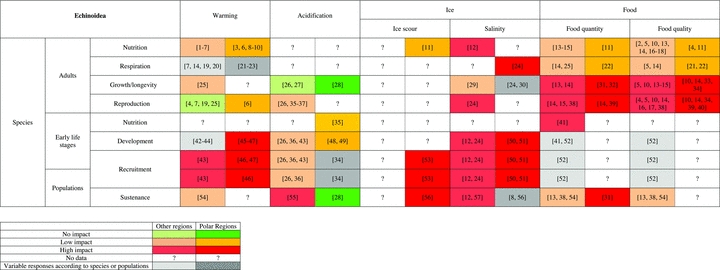

References: 1. Klinger et al. 1986; 2. Lares and McClintock 1991; 3. Lawrence and McClintock 1994; 4. McBride et al. 1997; 5. Otero–Villanueva et al. 2004; 6. Siikavuopio et al. 2006; 7. Siikavuopio et al. 2008; 8. Himmelman et al. 1984; 9. Miller and Mann 1973; 10. Vadas 1977; 11. Norkko et al. 2007; 12. Watts et al. 2001; 13. Andrew 1989; 14. Lawrence and Lane 1982; 15. McBride et al. 1999; 16. Beddingfield and McClintock 1998; 17. Klinger 1982; 18. Lawrence et al. 2009; 19. Percy 1974; 20. Ulbricht 1973b; 21. Brockington and Clarke 2001; 22. Brockington and Peck 2001; 23. Lee et al. 2001b; 24. Stickle and Diehl 1987; 25. Spirlet et al. 2000; 26. Dupont et al. 2010; 27. Shirayama and Thornton 2005; 28. Catarino et al. Submitted; 29. Lau et al. 2009; 30. Campbell and Russell 2004; 31. Blicher et al. 2007; 32. Lawrence 1975; 33. Daggett et al. 2005; 34. Russell 1998; 35. Dupont and Thorndyke 2008; 36. Kurihara 2008; 37. Siikavuopio et al. 2007; 38. Lamare et al. 2002; 39. Chiantore et al. 2002; 40. Moore and Manahan 2007; 41. Miner 2007; 42. Bingham et al. 1997; 43. Byrne 2010; 44. Fujisawa 1993; 45. Pearse et al. 1991; 46. Stanwell–Smith and Peck 1998; 47. Tyler et al. 2000; 48. Clark et al. 2009; 49. Catarino et al. 2011; 50. Cowart et al. 2009; 51. Sameoto and Metaxas 2008; 52. Vaitilingon et al. 2001; 53. Palma et al. 2007; 54. Ebert et al. 1999; 55. Hall–Spencer et al. 2008; 56. Scheibling and Hatcher 2001; 57. Andrew and Byrne 2001.

## Responses of benthic biota to environmental change

### Foraminifera

Foraminiferal assemblages in the waters around the Antarctic continent are likely to respond to many of the environmental shifts associated with climatic changes. In particular, species with calcareous tests will be disadvantaged by any shoaling of the CCD resulting from ocean acidification (see references Table 1). Based on a survey of records from the SO, Saidova (1998) concluded that carbonate dissolution is one of the principle factors influencing the distribution of these assemblages. At present, the depth of the CCD around Antarctica is highly variable, ranging from a few hundred meters on the shelf (Anderson 1975; Ward et al. 1987) to 4000 m or more in oceanic areas, such as the Weddell Sea (Mackensen et al. 1990; Dittert et al. 1999). The occurrence in some intrashelf basins, notably the bathyal Crary Trough (384–1079 m) in the southeast Weddell Sea, of foraminiferal assemblages consisting almost entirely of agglutinated species reflects the shallow CCD (∼550 m) in this part of the Weddell Sea (Anderson 1975). Similar predominantly agglutinated assemblages have been recognized at depths of 620–856 m and 79–796 m in the Ross Sea (Ward et al. 1987). We anticipate that such assemblages will become more widespread in the future.

Climatic changes may modify both the quantity and quality of organic matter fluxes to the seafloor. Such inputs, particularly of labile phytodetritus, exert a strong influence on the density and composition of foraminiferal assemblages (Altenbach et al. 1999; Loubere and Fariduddin 1999, Table 1) as well as the bathymetric distribution of particular foraminiferal species (De Rijk et al. 2000). Some deep-sea species bloom in response to seasonally pulsed phytodetritus inputs (Gooday 1988). These “phytodetritus species” (dominated by calcareous rotaliids) occur in the abyssal Weddell Sea where, as in the North Atlantic, they are often found living within phytodetrital aggregates (Cornelius and Gooday 2004). Indirect impacts arising from changes in the organic matter flux are also possible. A long time-series study (1989–2002) at the Porcupine Abyssal Plain (northeast Atlantic) has revealed decadal-scale trends in the abundance of some foraminiferal taxa, in addition to seasonal fluctuations (Gooday et al. 2010). One possibility is that these longer term changes are associated with sharp increases in the abundance of megafaunal holothurians, which, in turn, reflect changes in the quantity and quality of organic matter reaching the seafloor (Billett et al. 2010). It is possible that similar faunal shifts among benthic foraminifera will occur in the SO in future decades, as changes in the pH and temperature affect the composition of surface phytoplankton.

The disintegration of ice shelves, leading to a shift from an oligotrophic to a more eutrophic system in areas formerly covered by permanent ice, may affect foraminiferal community composition. Murray and Pudsey (2004) described “live” (rose-Bengal stained) and dead (unstained) foraminiferal assemblages from an area of seafloor to the east of the Antarctic Peninsula that previously lay beneath the Larsen Ice Shelf, which disintegrated in 1995. The samples were collected during the 1999–2000 and 2001–2002 seasons. “Live” foraminiferal densities in these samples were high, reflecting the high levels of primary production in the ice-free surface waters. Presumably, densities were lower prior to the ice shelf disintegration, although in the absence of baseline data from before the breakup of the ice shelf, this cannot be demonstrated. An important difference between “live” and dead assemblages is the higher proportion of agglutinated tests in the latter (43–98% compared to 25–66%). Since calcareous foraminifera are generally associated more closely with eutrophic conditions than agglutinated species, this could reflect an increase in surface primary production since 1995. Unfortunately, this interpretation, although appealing, is compromised by the likely postmortem dissolution of calcareous tests (Murray and Pudsey 2004).

The breakup of ice shelves and the consequential increased prevalence of drop stones may have either a negative or a neutral impact on many sediment-dwelling organisms, but it would provide sessile foraminifera with additional surfaces on which to live. Drop stones are often densely encrusted with these organisms. A total of 36 species (one calcareous and 35 agglutinated) have been recognized on drop stones from the abyssal northeast Atlantic (A. J. Gooday, unpubl. data). The *Discovery Reports* (Earland 1933, 1934, 1936) include 40 species, all of them agglutinated, that were found attached to stones and other hard substrates.

Finally, the effects of oxygen depletion on benthic foraminiferal assemblages will depend on the degree of oxygen depletion and whether or not it is permanent. Evidence from permanent oxygen minimum zones suggests that hypoxia will affect bathyal foraminifera species only when oxygen levels fall below a critical value, possibly 0.5 mL/L or less (Gooday et al. 2000; Gooday 2003; Levin 2003; Gooday et al. 2009; Table 1). Such concentrations possibly could develop in basins with restricted circulation. Species exposed to periodic (e.g., seasonal) hypoxia may be susceptible to less severe levels of oxygen depletion (Levin et al. 2009). However, these fluctuating conditions are usually associated with large rivers that disgorge large amounts of organic matter and nutrients onto continental shelves at lower latitudes. The most likely outcome in Antarctic waters is some diminution of oxygen levels that is not sufficient to affect benthic foraminifera.

### Nematoda

On the species level, information on nematode responses to environmental change for the SO is lacking, but experimental laboratory studies on species from coastal and estuarine areas in temperate regions indicate that rising temperatures, food quality and quantity, and salinity changes may have significant effects on the life history, reproduction, and feeding characteristics of many species (see Table 2, Forster 1998; Gerlach and Schrage 1971; Heip et al. 1978, 1985; Ishida et al. 2005; Kim and Shirayama 2001; Moens and Vincx 2000a, b; Pascal et al. 2008a, b; Price and Warwick 1980; Takeuchi et al. 1997; Tietjen and Lee 1972, 1977; Tietjen et al. 1970; Vranken and Heip 1986; Vranken et al. 1988; Warwick 1981; Wieser et al. 1974; Wieser and Schiemer 1977; Woombs and Laybournparry 1984). Even though the effect ranges tested in these studies go well beyond the expected environmental changes in the Antarctic and the magnitude of effects of similar temperature shifts may vary depending where along the temperature spectrum they occur, species responses are very likely under the predicted scenarios. A temperature increase of 2°C may shorten generation times, increase reproductive capacity and respiration, and result in a more opportunistic feeding behavior of certain nematode species with effects on the population level (Table 2 and references therein). It may therefore result in higher nematode activity and productivity, with pronounced dominance of certain species. Temperature changes and associated physicochemical modifications will affect nematode species differently, leading to imbalances on the community level. In the 1990s, an anomalous temperature drop of only 0.4°C in the Mediterranean deep sea caused a significant decrease in nematode abundance and functional diversity, concomitant with increased species richness and evenness (Danovaro et al. 2001; Danovaro et al. 2004). The small temperature shift allowed the community to change, possibly through migration of species. Even when normal temperatures returned, nematode diversity was only partially restored to previous values (Danovaro et al. 2001; Danovaro et al. 2004). It is therefore likely that deep-sea nematode communities in cold Antarctic waters will become much more affected by relatively small temperature changes. The same may hold true for shallow waters; phenological studies have indicated that nematode abundance and biomass decrease with increasing sediment temperatures (Yodnarasri et al. 2008).

Climate change-induced changes in density and composition of algae blooms may influence the quantity and quality of food that reaches the benthos (Hays et al. 2005; Smetacek and Nicol 2005). While food density is known to affect respiration, growth, reproduction, and feeding characteristics of certain nematode species (Table 2), the observed trophic plasticity of many nematodes prevents us from drawing conclusion on clear patterns. However, as a result of different species responses to changes in quality and quantity of food sources, population recruitment, structure, sustainability, and trophic interactions within the food web may be impacted and lead to changes in terms of nematode abundance, biomass, and structural and functional diversity. Indirectly, for instance, a rise in temperature may affect bacterial activity and decomposition rates, which in turn may affect trophic diversity in favor of bacteria-feeding nematodes. At the same time, it is important to realize that the investigated rates of (experimental) changes in food resources do not immediately fall within the expected ranges of climate change and severe impacts on species level are therefore not expected. Community shifts, however, are likely since changing food availability and quality will favor species equipped to exploit the new trophic conditions. Nematodes have been shown to feed on different food sources in Polar Regions implying selectivity in taking up and/or ingesting food in these areas (Moens et al. 2007; Guilini et al. 2010; Ingels et al. 2010; Gontikaki et al. 2011; Ingels et al. 2011). Very often, the more resilient and opportunistic nematode species that are able to feed on a variety of food sources and are less specialized become increasingly dominant in such a situation and may outcompete more specialized species with reduced diversity and evenness as a consequence.

Decreasing ice extent and density severely impacts nematode communities through increased iceberg disturbance and changes in food supply. Iceberg scouring (usually occurring on the shelf down to ca. 500-m water depth [Gutt et al. 1996; Dowdeswell and Bamber 2007]) can remove over 95% of the nematode community and cause a drop in diversity (Lee et al. 2001a, b). Although initial scouring has a deleterious effect, nematode abundance can recover within weeks. Scouring recovery occurs through recolonization, but without evidence for successional stages, suggesting that the nematofauna in frequently disturbed areas is well adapted (Lee et al. 2001b). Successional colonization and changes in nematode composition, however, are apparent in areas that have become ice free, such as the Larsen area at the Antarctic Peninsula (Vaughan et al. 2003; Raes et al. 2009a). Ice shelf collapse in this area has accelerated colonization of the new ice-free shelf areas because increased primary production at the surface is now able to supply the benthos with food. Nematode communities transformed after ice-shelf collapse from depauperated, low-diversity communities, to richer and denser communities dominated by opportunistic species (Raes et al. 2009a; Hauquier et al. 2011). In coastal areas, reduction of ice extent exposes the shallow waters and benthic environment to wind-driven currents and disturbance events, which may lower nematode abundance and diversity as has been shown in the Magellan area (Chen et al. 1999) and Arctic coastal areas (Urban-Malinga et al. 2004). At the same time increased production of macro-algae and phytoplankton may act to increase nematode densities and change community composition (Vanhove et al. 1998; Fabiano and Danovaro 1999; Vanhove et al. 2000; Skowronski and Corbisier 2002; Urban-Malinga and Burska 2009; Urban-Malinga et al. 2009).

In addition, increased benthic food deposition may lead to deoxygenation of the water through higher decomposition rates and increased respiration (Hofmann and Schellnhuber 2009). Among the meiofauna, nematodes are the most tolerant to low oxygen concentrations and may attain high densities and dominance (Neira et al. 2001a; b; Table 2; Gutierrez et al. 2008; Levin et al. 2009). Nevertheless, hypoxia in bottom waters may alter community composition by favoring those nematode species tolerant to low oxygen levels (Hendelberg and Jensen 1993). However, food availability has a greater impact on nematode communities than oxygen levels in surface sediments (Vanreusel et al. 1995). This is supported by Cook et al. (2000) who gave evidence that not severe hypoxia, but food quality was the main predictor of nematode abundance in the oxygen minimum zone of the Arabian Sea. Deoxygenation of Antarctic bottom waters may have severe consequences for benthic biota, with nematodes being less affected than other taxa. Community responses to hypoxia may therefore lead to a state in which nematodes are likely to be the dominant metazoan group.

Experimental studies investigating the effect of CO_2_ sequestration on meiofauna in the deep sea indicate that nematodes are sensitive to high CO_2_ concentrations in seawater (Table 2, Barry et al. 2005, 2004; Fleeger et al. 2006, 2010). Kurihara et al. (2007a) reported no lethal effects when pH was lowered with 0.80 units below normal (CO_2_ concentration of >2000 ppm above ambient). However, the effect that CO_2_ and pH have on deep-sea nematodes may depend on the type of source (Barry et al. 2005; Pascal et al. 2010). Other studies have reported that severe hypercapnia associated with pH levels of 5∼6 severely impairs the survival of nematodes, but also reductions in pH of only 0.2∼1.0 units below normal can result in high nematode mortality (Barry et al. 2004, 2005; Carman et al. 2004; Fleeger et al. 2006, 2010). The effects on nematodes, however, were less severe than for other taxa. These deep-sea studies suggest that “moderate” CO_2_ exposure, compared to the range of exposures possible following CO_2_ release, may impair survival in deep-sea nematodes (Fleeger et al. 2006). In contrast, CO_2_ effects on nematode communities from shallow-water micro- and mesocosm experiments pointed to high survival compared to other benthic metazoans. Drastic survival impacts only seem to occur under pH conditions of 5.5∼6 or less in a microplate study by Takeuchi et al. (1997) while in mesocosm experiments nematode diversity decreased, but abundance increased in response to realistic pH reductions mimicking ocean acidification predictions (Widdicombe et al. 2009; Hale et al. 2011). The diversity effects were of lower magnitude than for macrofaunal organisms, however, and abundance increases are likely the result of reduced predation and competition in the mesocosms. The studies mentioned here, suggest different effects on nematodes in shallow- and deep-water environments, but they also point out that the nematode community may become more dominant and less diverse in benthic ecosystems in response to ocean acidification.

### Peracarid crustaceans: amphipods and isopods

Both amphipods and isopods are marine ectotherms, which are generally considered to be among the most stenothermal organisms on Earth (Peck and Conway 2000; Aronson et al. 2007), and are characterized by slow physiological rates, growth, and great age (Wägele 1989; Peck and Brey 1996; Peck 2002; Held and Wägele 2005). Some eurytopic and opportunistic species exist in this group, but in general, amphipods and isopods are expected to show particular vulnerability to a change of conditions they are adapted to, and responses to rising temperatures are therefore expected on the species level. This is especially the case for the many brooding species because of their decreased migration potential, and hence reduced ability to disperse as an answer to a changing environment.

Research performed on the Antarctic amphipod *Themisto gaudichaudi* indicated that individuals living in warmer water exhibit an increased respiration rate, faster growth, earlier sexual maturity, and a smaller body size (Auel and Ekau 2009). These physiological features also have an impact on the feeding habits and requirements of the species. At higher temperatures, the increasing oxygen demand reduces the aerobic scope of animals (Peck 2002), and the demand for food will increase with increasing metabolic needs, leaving less resources for growth and reproduction. In turn, a smaller body size could limit the range of prey they are able to feed on and reduce their mobility. Moreover, smaller adult size and reduced mobility may negatively affect reproduction rates and increase predation risk to a point where predation losses may prevent survival of the population. At the same time, smaller individuals seem more tolerant to acutely elevated temperatures than larger individuals within the same species (Peck et al. 2009). It is likely that where warming is significant over monthly to annual time scales large individuals will be more affected than small ones, especially considering that thermal tolerance levels are lower under chronical temperature rises compared to acute temperature increases (Pörtner et al. 2007). The early loss of larger individuals will impact the population severely since they represent the major reproductive component (Peck et al. 2009). Sea water temperature increases of only a couple of degrees may hence affect peracarids’ physiology and are likely to modify drastically the distribution of *T. Gaudichaudi* and many other amphipod species (Maranhão and Marques 2003; Auel and Ekau 2009; Table 3). Such a selective removal of the larger individuals within a species will probably result in an ecological imbalance, with major consequences for the peracarid community as a whole (Table 3, 4). Temperature-dependent, selective removal will also be exhibited between peracarid species since temperature effects depend on the feeding behavior and activity of individual species. According to Clark and Peck (2009), very few Antarctic marine species are able to acclimate and perform normal biological functions over periods of months at temperatures above 4°C. Among Antarctic amphipod species, *Cheirimedon femoratus* can acclimate to 4°C (Peck et al. 2009) but the situation is complex in *Paraceradotus gibber*, there is an absence of classical heat shock response and the species is incapable of acclimatizing (Clark et al. 2008). Measuring the thermal tolerance limits of 14 Antarctic benthic invertebrates, Peck et al. (2009) found that the most active animals, three species of preying/scavenging amphipods in this case, exhibited higher tolerance to increasing temperatures than less active species. Such discrepancy between active groups, such as predators and juvenile individuals, and more passive organisms, such as sessile feeders, could have far-reaching consequences on the community level by disturbing the ecological balance and complexity.

For isopods, temperature has an effect on rates of transcription of several proteins in the muscles, including actin and myosin heavy chains, with increasing levels of expression as temperature increases in temperate and Antarctic species (for overview see Table 4). In the Antarctic *Glyptonotus antarcticus*, rates of protein syntheses were extremely low compared to the temperate isopod *Idotea rescata*. This was probably due to the relatively high energetic cost of protein synthesis for *G. antarcticus* in cold Antarctic waters in association with low rates of oxygen uptake (White 1975). An experimental study on the effect of temperature and salinity on vital biological functions (response to food odor, righting, swimming, and reburying) of the Antarctic isopod *Serolis polita* suggests that Antarctic isopods are vulnerable to environmental changes and their ability to cope with them is limited. Some biological functions (righting and burying) were more affected than others (swimming). Interaction effects between temperature and salinity showed that *S. polita* was more vulnerable to lower salinities when exposed to higher temperatures (Janecki et al. 2010). The predicted higher temperatures and concomitant decrease in salinity may therefore affect isopod survival to a greater extent than originally thought. Salinity change in itself does not seem to have a strong effect on isopods, but there is some evidence that isopod populations from intermediate salinities were more polymorphic than populations from extreme salinities (Heath 1975). However, recent investigations of physiological responses to salinity changes of the isopod *I. chelipes* from the Baltic brackish waters documented that osmotic adjustment may be more or less costly in terms of energy according to salinity (Lapucki and Normant 2008).

The outcome of global change effects on the survival of individual organisms or populations will not be dictated by its physiological limits, but by ecophysiological constraints on its capacity to perform critical biological functions, such as locomotion and feeding (Pörtner et al. 2007). A temperature effect on motility (walking and righting) of Antarctic crustaceans compared to temperate species (Young et al. 2006) showed that even though Antarctic species have a lower thermal dependence, the thermal scope within which they can perform biological functions is reduced compared to temperate species. This implies that Antarctic peracarids are very much adapted to the narrow, cold temperatures, but also that they are much more vulnerable to aberrant temperature changes than their temperate relatives.

Despite the lack of calcium carbonate in the exoskeleton of amphipods and isopods, implying that lower pH values and shoaling of the CCD would not affect their structural development, ocean acidification presents a real threat to Antarctic peracarids. Several studies (Kurihara et al. 2004a,b; Spicer et al. 2007) have shown that acidification will not affect crustaceans in terms of developmental success to the same extent it will affect bivalves (Kurihara et al. 2007b) or sea urchins (Havenhand et al. 2008), but it would certainly retard their embryonic development (Egilsdottir et al. 2009) and in synergy with other factors, such as reduced salinity, it can reduce the number of hatchlings (Vlasblom and Bolier 1971; Egilsdottir et al. 2009). For the isopod *G. antarcticus,* haemolymph pH values between 7.85 and 8.2 have been measured. Acid–based changes due to respiratory adjustment are poorly buffered in *G. antarcticus* due to the low protein buffering capacity of the haemolymph, implying that it is unable to compensate for temperature changes (Jokumsen et al. 1981). Therefore, species being affected would probably migrate to more favorable environments or suffer removal from the ecosystem in case such migration is unfeasible.

Climate change has affected crustaceans, including isopod and amphipod species, in the past. While the cold Antarctic temperatures pose limits to performance that exclude modern predators and circulation patterns form physical barriers preventing invasion from more northern latitudes, global warming is now slowly removing the barriers posed by cold temperatures and circulation patterns, enabling higher trophic level predators such as crabs, durophagous bony fish, or sharks (Aronson et al. 2007; Smith et al. 2011) to invade the Antarctic and influence the often indigenous character of its marine life. Mouritsen et al. (2005) showed that a 3.8°C increase in ambient temperature of the Wadden Sea is likely to result in a parasite-induced population collapse of the widespread amphipod *Corophium volutator* by increasing the transmission rate of their microphallid trematode parasites. Although this study is based on a North Atlantic species, one can easily envisage such a threat to SO amphipod species. Increasing rates of invasion, predation and/or competition, and increased risk of parasitism caused by climate change could not only affect the sustainability of certain species, it may disturb and alter amphipod species distribution and benthic community composition.

In analogy, following the Cretaceous extinction of Decapoda, the isopod families Serolidae and Antarcturidae radiated on the SO continental shelf, indicating successful diversification after reinvasion. In contrast, a genetic population study performed by Leese et al. (2008) showed that there is currently no effective gene flow for the species *S. paradoxa* between Patagonia and the Antarctic Peninsula and that a genetic connection has been absent for time exceeding the last glacial maximum. The authors argue that specimens from the Strait of Magellan and the Falkland Islands very likely represent two distinct species that separated in the mid-Pleistocene (about 1 million years ago) (Leese et al. 2008). Due to their size of few millimeters up to a few centimeters in the deep sea, the brooding and usually less-mobile isopods (excluding Munnopsidae) are thought to have a reduced gene flow. However, even though isopods are not very mobile, they may respond with migration to climate change nowadays (Barnes et al. 2009b), especially in the SO deep sea where 50% of all Isopoda sampled during the ANDEEP expeditions are Munnopsidae (Malyutina and Brandt 2007) that can swim. However, besides their migration potential, Isopoda must also have an ability to adapt to changing environments because they successfully colonized all marine environments from the tropics to the poles and from the shelf to the deep sea; the deepest records of the family Macrostylidae are from > 10,000 m (*Macrostylis galatheae*Wolff 1956 from the Philippine Trench). It is therefore considered unlikely that extinction will occur in Isopoda due to climate change. However, at local scales, global change effects may affect individual species, generating selection pressures that favor more tolerant species or ecological groups over more vulnerable ones. Benthic isopod assemblages are therefore likely to change and this might also affect species’ vulnerability on longer time scales.

Quantity and quality of food is important for all animals, especially early developmental stages, but Isopoda are brooders and at least the offspring or early developmental stages are relatively independent from food input. However, it is known that the SO Isopoda have larger eggs than their boreal and tropical relatives (Wägele 1987, 1988; Wägele 1989), and variability in food resources may affect their ontogeny (Table 4). A recent study reported that, generally, SO isopods utilize a diverse food spectrum, including phytodetrivory and carnivory, while certain munnopsid isopod species may prefer foraminiferans as food source (Würzberg et al. 2011a). Consequently, changes in isopod assemblages may translate into changing foraminifera communities. Amphipods have colonized a wide variety of ecological niches and have developed a large range of feeding strategies (Dauby et al. 2001). Many amphipods have a broad-spectrum diet, are not selective in prey–predator relationships, and take advantage of different food resources (Nyssen et al. 2002), although they are thought to be mainly carnivorous (Würzberg et al. 2011a). These opportunistic species are not likely to be severely affected by changes in food quality and quantity, although a moderate impact is expected (Table 3). In contrast, some amphipod species are highly specialized in food foraging, such as micrograzers feeding on a single food item; for example species of the genus *Echiniphimedia* feed exclusively on sponges (Nyssen et al. 2005). For such species, a change in food availability can have severe consequences on their sustainability in the long term. Shifts in food quality and quantity may therefore affect different species differently and shifts in community composition are likely. Since foraminifera are important for the diet of isopods, a shift in foraminiferan abundance would ultimately also affect the abundance and composition of Isopoda (Würzberg et al. 2011a).

### Echinoids

Seawater temperature rises, salinity drops, changes in food resources, and seawater acidification have been documented to affect Antarctic echinoids during some stages of their life cycle. As juveniles and adults, echinoids are epi- or endofaunal benthic organisms while their earlier developmental stages are pelagic (broadcasting species) or benthic (brooding species) (Pearse et al. 1991; Poulin and Feral 1996), and responses will depend on their respective biology and physiology. Byrne (2010) reported the variability of responses of echinoid embryos and larval stages to thermal and pH stressors, even for closely related species. Having a high-Mg calcite skeleton, echinoids may be particularly vulnerable to changes of the aragonite saturation horizon in the SO (Sewell and Hofmann 2011).

Seawater temperature rise in the Antarctic surface waters of 2–4°C in the next 100 years may have only minor impacts on the metabolic activities of postmetamorphic echinoids (Table 5). This is documented for *Sterechinus neumayeri* in the Antarctic (Belman and Giese 1974; Brockington and Clarke 2001; Brockington and Peck 2001), and is supported by several acclimation experiments using tropical (Klinger et al. 1986; Lares and McClintock 1991; Ubaldo et al. 2007) and temperate shallow water (Ulbricht 1973b; Siikavuopio et al. 2006; Siikavuopio et al. 2008; Lawrence et al. 2009), but also deep-water species (Ulbricht 1973a). Contrary to adults, juvenile forms may be more vulnerable to seawater temperature rise as indicated by studies carried out on early life stages of *S. neumayeri* (Stanwell–Smith and Peck 1998). This shallow-water species has planktotrophic pelagic larvae (Bosch et al. 1987). Gamete release coincides with the austral summer (Freire et al. 2006) and embryonic and larval development has an optimal window between 0.2°C and 1.7°C outside that both can be impaired (Stanwell–Smith and Peck 1998). Little is known about salinity effects on adult Antarctic echinoids, but there are indications that echinoderm metabolic rates are not affected when exposed to salinities within their tolerance range (Farmanfarmaian 1966). In fact, within acclimated sea urchin populations, adults show a much greater tolerance to lower salinities than juveniles (Himmelman et al. 1984). The influx of freshwater from melting ice shelves due to global warming can result in a bottleneck of larval recruitment, as salinity drops of only 2–4 units below normal slow down development rate and reduce developmental success of *S. neumayeri* embryos (Cowart et al. 2009).

Antarctic postmetamorphic echinoids are opportunistic feeders, allocate little energy to feeding and are able to react rapidly in the presence of sporadic nutrients (Lawrence and Lane 1982; Andrew 1989; Lawrence and McClintock 1994). Together with the fact that a large range of food items is used by each species (Lawrence 1975; De Ridder and Lawrence 1982; McClintock 1994; Jacob et al. 2003), this suggests that Antarctic echinoids would be able to acclimatize to changes in food resources, that is, to changes of the benthic components they rely on, such as preys and algae, as a result of seawater temperature rise. Trophic flexibility has been demonstrated for *S. antarcticus* in the Weddell Sea (Raes et al. 2009b), and for *S. neumayeri* in the Ross Sea where the individuals showed a shift from feeding predominantly on detritus (locations with more permanent sea ice in the South) to feeding on more freshly produced algal material (proximity to ice-free water in the North and East) (Norkko et al. 2007). Interestingly, all Antarctic species recurrently ingest detritus. According to Norkko et al. (2007), such a detrital pathway may reduce the impacts of large seasonal fluctuations in the availability of primary production. However, long-term consequences of dietary shifts on echinoid populations are complex to predict because of reciprocal effects between different stages of the feeding process that can vary between species. Independently from seawater temperature, the quality and quantity of the ingested food can influence each feeding step, going from ingestion to nutrient allocation to either somatic or gonadic growth, but, in turn, the size of the individual (resulting from somatic nutrient allocation) and its reproductive status (resulting from gonadic nutrient allocation) can also influence the feeding steps (Lawrence 1975; Lawrence and Lane 1982; Beddingfield and McClintock 1998; Otero-Villanueva et al. 2004). This is well documented in aquaculture studies (Russell 1987; McBride et al. 1999; Otero-Villanueva et al. 2004; Daggett et al. 2005; Siikavuopio et al. 2006; Siikavuopio et al. 2008), and for the Antarctic species *S. neumayeri* (Brey et al. 1995; Brockington and Clarke 2001; Chiantore et al. 2002) and *S. antarcticus* (Brockington and Peck 2001). Data concerning global-change effects on premetamorphic stages are scarce as indicated in Table 5, especially for the effects of diet quality on the development of planktotrophic larval stages. According to Marsh et al. (1999), feeding larval stages of *S. neumayeri* are not dependent on phytoplankton availability to complete their early development (up to day 60), and the uptake of dissolved organic matter by embryos and larvae could compensate for a scarcity of particulate food sources. However, food quality and quantity is known to influence greatly the survival, growth, and developmental success in larvae as well as metamorphosis and postlarval development in temperate and tropical species (Vaitilingon et al. 2001). Clearly, more research on Antarctic species is needed.

Adult sea urchin mortality does not seem to increase when exposed to lower pH waters, but their gonad growth can be affected (Siikavuopio et al. 2007; Kurihara 2008). Unfortunately, impacts of ocean acidification on adult Antarctic echinoid physiology are unknown and require further study. Interestingly, the spines of *Ctenocidaris speciosa*, (Weddell sea), which are lacking an epidermis and are hence directly exposed to physical and chemical conditions of seawater, showed adaptations that provide them with an advantage in acidified deep-sea environments (Catarino et al. Submitted). Although fertilization success and early embryogenesis stages of the Antarctic species *S. neumayeri* were demonstrated to be relatively robust to lowered pH (Ericson et al. 2010), the endotrophic larval development was significantly delayed at pH 7.6, a value expected to occur by 2100 (Clark et al. 2009; Dupont et al. 2010). Similarly, the larvae of the Antarctic and sub-Antarctic species *Arbacia dufresnei* suffered a larval development delay at pH 7.4 (Catarino et al. 2011). In both species, no significant increase of abnormal forms was recorded. It is worth mentioning that seawater pH reductions within the range of future predictions impair the larval development of *S. neumayeri* less than for temperate and tropical species (Clark et al. 2009). On one hand it is possible that sea urchins from naturally stressful environments can cope better with a changing environment. On the other hand, slower metabolism rates can improve resistance to hypercapnia (Pörtner 2008). These results should be interpreted with caution since little information is available on the effects of low pH on the exotrophic larval stage or on metamorphosis processes. Surprisingly, exposure to pH 7.7 was reported to increase the number of successfully metamorphosed juveniles of *Strongylocentrotus droebachiensis,* although these were smaller than juveniles developed at control pH and took more time to complete their development (Dupont and Thorndyke 2008). Furthermore, temperature and pH may have interactive effects on sea-urchin development as documented for nonpolar species (Byrne 2011).

Early echinoid life stages are particularly sensitive to stressors and perturbations (Pörtner and Farrell 2008; Melzner et al. 2009; Byrne 2010), making them vulnerable in terms of recruitment success and long-term viability of populations (Morgan 1995; López et al. 1998). Under the predicted environmental change, one of the main challenges for the future of Antarctic echinoid populations will be the ability of echinoids to successfully complete their development. Impairment of gonad development or gamete quality in adults could further affect reproduction and recruitment processes. In general, information on the long-term effect of stressors (temperature, diet shifts, pH) is lacking (Table 5) and consequently the viability of echinoids populations in response to global change remains difficult to assess.

## From individual to ecosystem responses

Most information on the physiological ability of individuals and species to cope with environmental change pertains to organisms within the macro- and megafauna size range (amphipods, isopods, and echinoids, see Tables 3–5). They show that certain species are adapted to the cold temperatures of the SO, and that such adaptation has rendered many of them very sensitive to temperature changes. This is especially the case for larger, older, and less-active species rather than smaller, younger, and more active species such as predators and scavengers (Peck et al. 2009). However, in the case of echinoids, early life forms are expected to be more vulnerable than adults (Belman and Giese 1974; Stanwell-Smith and Peck 1998; Brockington and Clarke 2001; Brockington and Peck 2001). In general, the predicted temperature increases are large enough to exceed the physiological capacities of many stenothermal organisms, and the fast rate of change may imply that these organisms will not be able to migrate or adapt within the time available to do so. This is especially the case for animals exhibiting brooding, such as many isopods, because of their limited migration potential. Species extinctions are likely to occur as environmental change goes beyond the window within which physiological processes or ecophysiological actions can be performed. Extensive extinctions during the Pleistocene among some deep-sea foraminiferal taxa (*Stilostomella* extinction) seem to have been linked to environmental changes associated with cooling events (Hayward 2002). In addition, temperature changes act in synergy with processes influencing oxygen metabolism, that is, when temperatures are raised the capacity to take up oxygen is often limited at a cellular level (Pörtner 2001, 2010). This illustrates that it is not just the cost of compensating for temperature changes; it may also impede an organism's ability to take up oxygen and preclude survival of the individual. For the smaller organisms such as foraminiferans and nematodes, likely individual or species responses to predicted temperature changes are usually limited to an increase or decrease in the rate of performing (eco)physiological functions without threatening the individual species or populations, but information for Polar species is generally lacking, as indicated by the lack of data references in Tables 1–5. However, a maximum rise of 2°C compared to current temperatures, as predicted by 2100, is not expected to remove species, although it may alter community patterns through shifts in dominance and trophic composition in favor of the more resilient species.

Ocean acidification will affect a large range of species, especially those depending on calcium carbonate for growth of their shell or skeleton. For the Foraminifera, knowledge is lacking about the effects of shoaling of the CCD and lowering of seawater pH on their physiology. However, calcareous taxa are largely absent below the CCD in oceanic environments. These changes therefore would probably lead to the removal of calcareous species and hence to communities dominated by agglutinated foraminifera (Saidova 1998). Physiological foraminiferal responses to ocean acidification have not been documented. In the case of nematodes, mortality at the community level follows declines in pH (Barry et al. 2005; Fleeger et al. 2006, 2010), but information on nematode species-specific responses is absent. In shallow waters, nematodes may display lower sensitivity to realistic future OA conditions compared to other taxa, but their diversity is likely to be affected. Despite the lack of calcium carbonate in the exoskeleton of isopods and amphipods, OA may affect their embryological stages and reduce the number of offspring (Egilsdottir et al. 2009). For echinoids, OA may affect adults and larval or embryonic stages differently (Catarino et al. 2011; Dupont et al. 2010; Byrne 2011), but variable results indicate the need for further study. In general, studies suggest that even though adults may have the capacity to cope with certain levels of OA, producing offspring may be impaired and lead to a reduced recruitment in postmetamorphic populations, although the contrary has also been documented. Taking into account the high vulnerability of other benthic groups such as bivalves and cold-water corals, OA will promote the removal of sensitive species, but variable responses between groups imply a distortion of the ecological balance of the ecosystem.

The presence of ice in the marine and terrestrial environment in the Antarctic influences the fauna substantially. Initially, increased iceberg scouring as a result of rising temperatures and subsequent collapse of ice shelves and glaciers, may not affect the physiology of organisms, but it will have drastic local impacts at the community level with recurring removal of a large fraction of the benthic community (Gutt et al. 1996; Gutt and Starmans 2001; Lee et al. 2001a, b; Gutt and Piepenburg 2003). In the longer term, the disappearance of seasonal ice coverage and glaciers may reduce diversity by lowering the frequency of iceberg disturbance events that help to maintain this (Gutt et al. 2011). An increase or reduction in iceberg density may cause drastic change in pelagic and benthic food webs and their coupling, considering the chemical and biological enrichment associated with icebergs (Smith Jr et al. 2007; Smith Jr 2011). Ice-shelf collapse may expose for the first time large areas of seafloor to phytodetrital input, instigating colonization processes and changing communities (Raes and Vanreusel 2005; Gutt et al. 2011; Hardy et al. 2011). On the other hand, the melting of ice will lead to salinity changes as a result of reduced brine rejection and increased fresh-water flow, with effects on the (eco)physiology and survival potential of marine organisms (Cowart et al. 2009; Janecki et al. 2010).

Changes in the quality and the quantity of the food that reaches the benthos are likely for certain taxa. Depending on the food-requirements of the species, these changes may render certain trophic groups more vulnerable than others. Among foraminifera, species associated with more eutrophic conditions are likely to replace those found in oligotrophic settings. They may include “phytodetritus species” that flourish where the supply of phytodetritus is seasonally pulsed. Nematodes are also sensitive to changes in food supply, with effects on respiration, growth, reproduction and feeding processes, but also community changes in favor of the more opportunistic or well-adapted species. Changes in food supply may affect isopods, amphipods, and echinoids that feed on specific food sources, but opportunistic species displaying trophic plasticity are likely to be less sensitive.

One likely response to climate change will be species migrations (Barnes et al. 2009b). Failing this, species will either become extinct or be forced to adapt to, or at least tolerate, the new conditions. Which of these responses occurs will depend on local conditions, the community interactions, and the species vulnerability to any of the environmental perturbations. Temperature rises within the range predicted may be responsible for migrations and invasions of species into new habitats, which were previously unsuitable for the survival of those species. Subsequently, the new arrivals may increase competition pressure on the original residents, which are already trying to cope with new physiological demands. Such invasions have been observed in the marine Antarctic (Thatje and Fuentes 2003; Clarke et al. 2005; Thatje et al. 2005; Smith et al. 2011) and although the full range of effects on the local populations remains unclear (Thatje 2005), major ecological impacts are likely (Smith et al. 2011). The ecological imbalance following species migrations may also lie in the fact that certain functional groups, such as brooding species, have limited dispersal capacities and hence limited potential to avoid unfavorable environmental conditions.

The response of organisms to a changing environment depends on their capacity to cope with the physiological cost imposed by the new conditions (Peck 2004, 2005). In such a situation, individuals have a limited number of responses that enhance survival in changing environments. They can (1) acclimatize using their physiological flexibility and capacity to sustain new biological requirements, (2) adapt to their new environment within the constraints imposed by their reproductive capacities and genome, (3) migrate to locations where conditions remain within their physiological range, or (4) suffer extinction by failing to cope, adapt, or migrate (Barnes et al. 2009a). The cost that a changing environment poses varies from one species to another depending on their biology, physiological adaptations, and dispersal capacities. These factors will ultimately decide the nature of their response. A recurrent observation is that the impact of environmental changes at the physiological and individual level, which is likely to result in changes at the community and ecosystem levels, is variable, even between different life stages of the same species. Unfortunately, there is still a poor understanding of the mechanisms underlying observed environmental change effects at the individual and taxon level. Such knowledge is crucial, because it may lead to a better understanding of generalizable mechanisms with applicability across species and communities.

At the level of populations, the outcome of change is determined by the population's ability to sustain itself. Individuals may be able to cope physiologically, but reduced genetic connectivity between populations caused by hydrodynamic changes, environmental shifts changing the boundaries of physiological sustenance, and biological alterations may change species distributions and/or reduce or eliminate populations, which, in turn, can enforce future speciation. Shifts in species and trophic assemblages, species extinctions, migrations, changes in food supply may instigate drastic changes in food webs on an ecosystem scale and affect ecosystem functioning. At the community level, a broad range of biological interactions increases the uncertainty of predicting ecosystem responses to climate change effects. Without a comprehensive understanding of the ecofunctional role of taxa within a complex and interactive ecosystem, an overall understanding of how ecosystems and communities will respond to environmental change is unlikely. Crucial in pursuing such knowledge is gaining insight in the complex set of trophic interactions and cascading mechanisms between organisms (across life-stages and taxa) that are contained within ecosystems, such as predator–prey relationships and competitions. Such an approach asks for specific considerations when tackling climate change effect questions on ecosystems, whereby species with important ecological roles should be identified, as well as the key interactions between these species and the essential components of their broader ecosystem (Russell et al. 2011). An integrated approach including macro-ecological concepts, experimental evidence, modeling approaches with energy budgets incorporated in life cycle models, and attention for the effect-mechanisms and organism or life-stage interactions is crucial in identifying and predicting ecosystem level changes in response to climate change (Russell et al. 2011).

This review has highlighted our lack of understanding of climate change effects on selected benthic taxa at different levels of biological organization, in particular for the SO (Tables 1–5). In the case of the meiofauna, we are only starting to appreciate the effects of climate change on physiological processes and population sustainability. Most studies have investigated the sensitivity of taxon-specific communities to environmental change without addressing trends and processes at an individual or species level. For the peracarid crustaceans (isopods and amphipods), some recent experimental studies have yielded insights into the effects of warming and acidification on individuals and species (see Table 3, 4). Our understanding of effects at the taxon-specific community level, however, remains poor. Echinoids are a good example of how experimental studies can reveal climate change effects on the physiology of individuals and species. Again, however, little is known about effects on their communities. These gaps in our knowledge prevent us from understanding how observed physiological effects influence the sustainability of populations and communities. The inadequate understanding of ecosystem sensitivity to climate change is exacerbated by the lack of information about interactions between these different levels of biological organization, as well as between different taxa. Experimental and modeling approaches that yield better data regarding niche-exploitation and food-web and energy dynamics and other interspecific interactions should improve our ecosystem-level understanding. Another problem is the lack of data on Antarctic organisms. Much more information is available from temperate environments, but this is difficult to extrapolate to Polar habitats. In general, there is a crucial need for studies on the physiology, behavior, taxonomy, biogeography and community interactions of organisms in the SO if we are to understand the full repercussions of anthropogenically induced climate change on sensitive Antarctic ecosystems.

This study has identified the extent of our knowledge about the effects of climate change on five important zoobenthic groups in the Antarctic, but has also exposed our lack of understanding of how the SO benthic ecosystem will respond to climate change. There is an urgent need for additional research aimed at clarifying this crucial issue.

## Future Research Goals and Recommendations

- Extension of analyses of sensitivity to other important benthic taxa, for example, microbiota, polychaetes, molluscs, sponges, and other groups of echinoderms.
- Experimental studies on physiological and population-level responses of additional taxa to warming and acidification of the oceans.
- Integrated biological research ranging from multitaxon physiological sensitivity studies up to community and ecosystem-based research, and the integration of interactions between taxa and functional groups into modeling studies.
- Increased efforts to realize reliable environmental niche models to project species’ currently realized environmental niches onto future climate change scenarios.
- Enhanced support for biodiversity studies dealing with functional aspects of biodiversity, including comprehensive phylogeographic and population genetic studies with links to ecosystem functioning.
- Surveys of community composition and structure below permanent ice shelves, in order to provide baselines for studies of faunal change following any future ice-shelf collapses.
- The establishment of marine protected areas in the SO, especially on the deeper shelf, and at bathyal and abyssal depths where climate impacts are thought to affect communities in the near future.
